# Twenty-Year Survival of Kidney Transplant From a Deceased Donor With Autosomal Dominant Polycystic Kidney Disease

**DOI:** 10.1016/j.ekir.2021.05.030

**Published:** 2021-06-10

**Authors:** Naim Issa, Maroun Chedid, Maria V. Irazabal, Patrick G. Dean, Fouad T. Chebib

**Affiliations:** 1 Division of Nephrology and Hypertension, Mayo Clinic, Rochester, Minnesota, USA; 2 Department of Transplantation Surgery, Mayo Clinic, Rochester, Minnesota, USA

## Introduction

Kidney transplantation remains the best modality for renal replacement therapy for patients with end-stage kidney disease. It is well established that kidney transplantation confers to those patients better long-term survival and quality of life than remaining on the waiting list or chronic dialysis.^1^^,^^2^ In the United States and worldwide, there is a significant shortage of organs. For that reason, the donor deceased pool has been expanded to increase the supply of kidneys to account for that high demand for kidney transplantation. Autosomal dominant polycystic kidney disease (ADPKD) is the most common cause of inherited cystic kidney diseases and affects at least 1 in every 1000 individuals worldwide.^3^ ADPKD is characterized by slow progression of cyst growth along with the slow deterioration of renal function. Previously published data from case reports of patients are limited but show good outcomes, at least in the short-term.^4^ We present herein a case of a kidney transplant recipient who has ADPKD who received a directed deceased donor kidney transplant from a related young donor who has the same ADPKD mutation and had great long-term kidney function despite cyst progression. The recipient passed away with a functional kidney transplant after 20 years.

## Case Presentation

**Figure 1:**
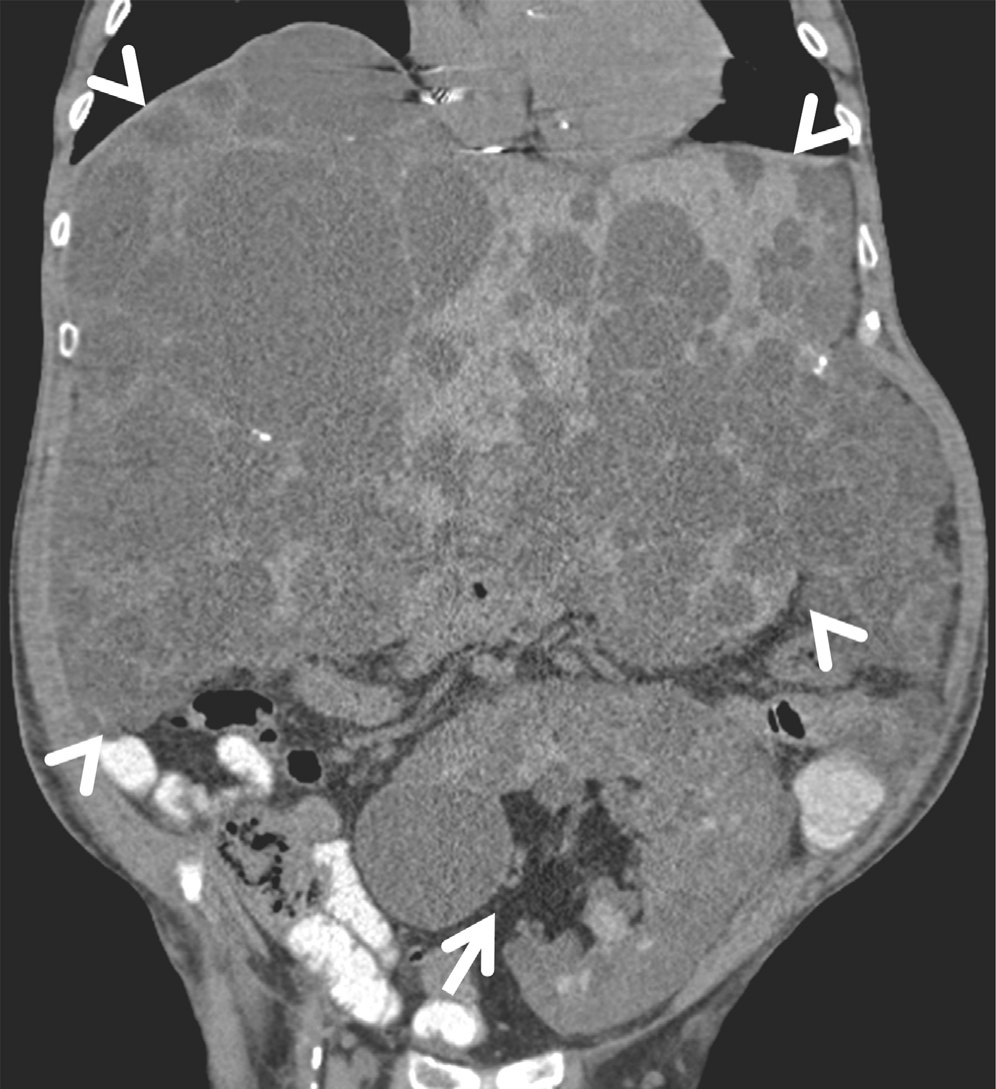
A computed tomographic scan of the abdomen without intravenous contrast shows severely enlarged polycystic liver (arrowheads) and a polycystic kidney transplant in the left lower quadrant (arrow). The cystic allograft volume is 1244 ml.

**Figure 2:**
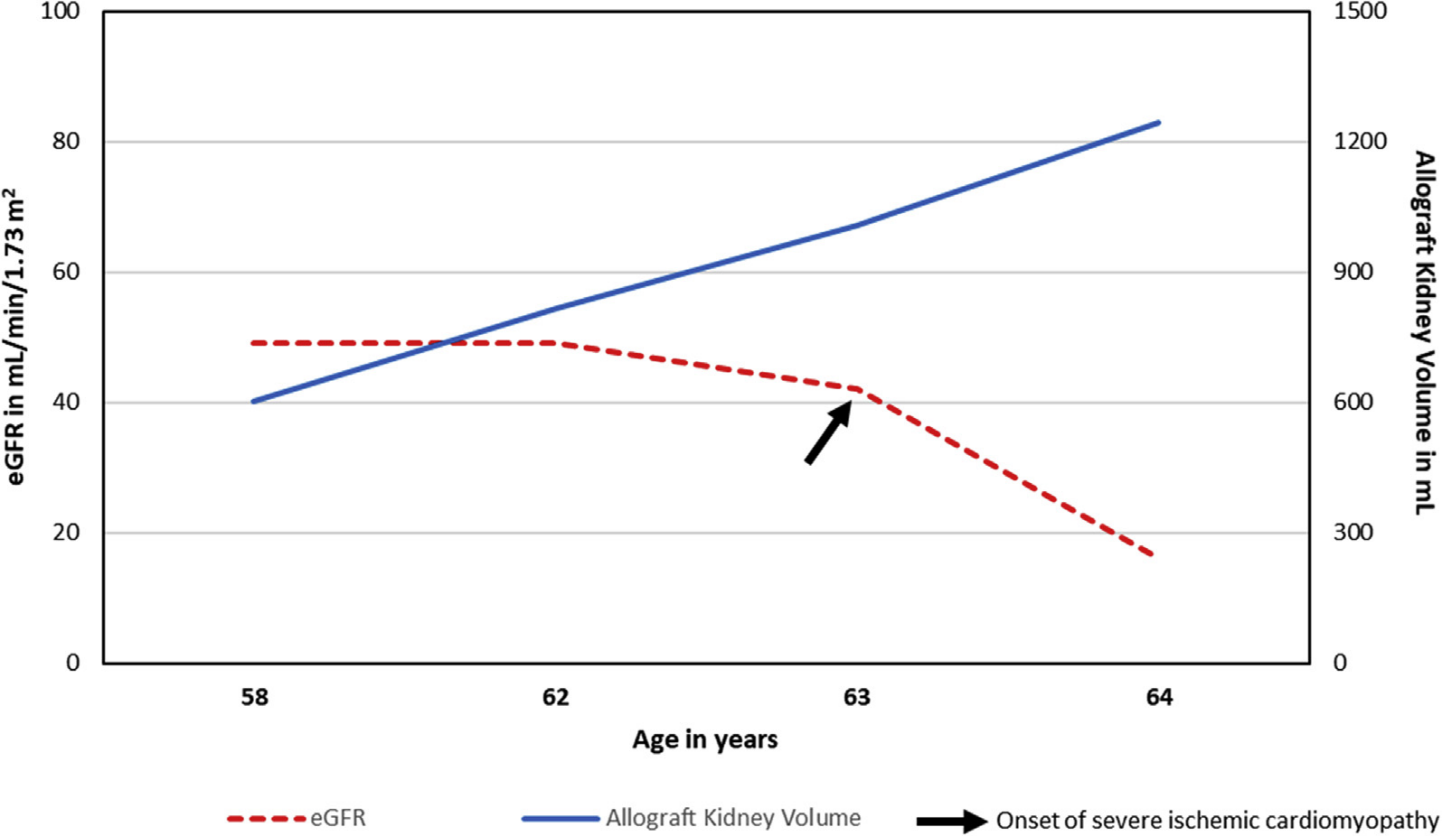
The graph depicts the trend of estimated glomerular filtration rate and allograft kidney volume through time in the preceding 6 years before the patient’s death (338 × 190 mm [300 × 300 dpi]).

We present a case of a 44-year-old man with end-stage kidney disease from ADPKD due to a frameshift truncating mutation in the *PKD1* gene. After being on hemodialysis for less than a year, he received a directed related deceased donor kidney transplant from a 21-year-old male first cousin known to have ADPKD and who died from a motor vehicle accident. The donor/recipient human leukocyte antigen mismatch was reported as 3/6. The recipient received induction immunosuppression with corticosteroids (only induction agent available at that time). Ten years later, the recipient underwent a right native nephrectomy followed by a left nephrectomy for significantly enlarged and symptomatic kidneys as well as a significantly enlarged polycystic liver. Over the course of 20 years, the recipient was maintained on cyclosporine and prednisone and enjoyed a stable renal allograft function with a serum creatinine of 1.5 to 1.7 mg/dl without any episodes of rejections or urinary tract infections. His renal allograft function declined rapidly with several episodes of acute kidney injury in the setting of new-onset severe ischemic cardiomyopathy with a reduced left ventricular ejection fraction of 36% and severe mitral valve regurgitation. He also developed significant hypervolemia and ascites. At that time, his serum creatinine was as high as 3.2 to 4.1 mg/dl in the setting of aggressive use of a combination of thiazide and loop diuretics along with aquapheresis. He was quite symptomatic due to the enlarged cystic liver. In addition, he had significant abdominal fullness and ascites contributed partially by the cardiac failure. The distribution of the cysts in the liver was such that he was not a candidate for aspiration foam sclerotherapy, cyst fenestration, or surgical resection. He was treated conservatively for his heart failure due to the underlying comorbidities, including severe polycystic liver disease. To improve his kidney allograft function, the cyclosporine was switched to sirolimus without improvement of his kidney allograft function. He subsequently died at the age of 64 years from complications related to decompensated heart failure. A computed tomographic scan of the abdomen without intravenous contrast (Figure 1) shows severe polycystic liver disease (arrowheads) and a polycystic kidney transplant in the left lower quadrant (arrow). The cystic allograft kidney volume was 1244 ml with a growth rate of 19% per year over the preceding 6 years. The trend of the kidney allograft volume along with the estimated glomerular filtration rate over the preceding 6 years is depicted in Figure 2.

## Discussion

Despite the *PKD1* truncating mutation, this kidney allograft lasted for 20 years. It likely would have lasted longer because the patient died with a functioning kidney allograft from complications related to heart failure. In the United States, ADPKD affects around 600,000 individuals, accounting for 5% of patients with kidney failure. The median age of reaching kidney failure in ADPKD patients is 54 years.^5^ Some with asymptomatic ADPKD may die of unrelated causes (e.g., motor vehicle accidents), and their kidneys may be offered for donation. With the shortage of organs worldwide, transplanting an ADPKD donor kidney from a young deceased donor with normal renal function in selected recipients can be considered. It is essential to highlight that an ADPKD deceased donor’s age remains crucial when considering accepting such an organ. In our case, this was a directed deceased donation from a first-degree cousin, which is a rare occurrence. The following criteria for accepting ADPKD deceased donors were proposed by Olsburgh *et al.*^6^: (i) age less than 50 years, (ii) kidney size of less than 15 cm, (iii) normal creatinine at the time of procurement of the kidney, and (iv) a predicted cold ischemic time preferably of less than 12 hours.

Assessing the risk of cystic disease progression in the deceased donor is essential in making the decision. A practical approach is to use the age-adjusted total kidney volume represented by the Mayo imaging classification.^5^^,^^7^ This classification allows to predict the intrinsic rate of growth of the cystic kidneys and future kidney function decline. Patients with Mayo imaging class 1A and 1B are slow progressors with a mild decline in renal function in the first 4 decades of life.^8^ Kidneys from deceased donors with class 1A or 1B would be expected to have a low cystic burden and slow renal function decline.

Transplanting polycystic kidneys presents numerous challenges, including difficulty to biopsy, continuous cyst growth, stone formation, and risks of cysts bleeding and infections. For that reason, it is essential that the recipient is thoroughly counseled and can give informed consent before receiving a polycystic kidney. In the context of the current severe shortage of organs, this case highlights the possibility of successfully transplanting polycystic kidneys from young deceased donors with normal renal function, thereby expanding the pool of deceased donor kidneys. It also highlights that those selected polycystic kidneys can have excellent long-term allograft outcomes despite kidney cyst growth. Moreover, those polycystic kidneys could be offered to older (>65 years), well-informed recipients. We propose the following criteria for accepting such donors: (1) donor age <40 years, (2) normal renal function at the time of death, and (3) Mayo imaging class 1A or 1B (Table 1).

**Table 1:** Teaching points

1. A significant shortage of kidney organs in the United States and worldwide remains a dire need for kidney disease patients awaiting kidney transplantation.
2. ADPKD is highly prevalent and characterized by kidney cysts’ growth and deterioration of renal function.
3. Polycystic kidneys can be offered to older and well-informed recipients from suitable deceased donors to expand the pool of donors and address the organ shortage.
4. Polycystic kidneys can have excellent long-term allograft outcomes despite the kidney cystic continuous growth.
5. The proposed acceptance criteria for ADPKD kidneys from deceased donors are as follows: (1) donor age <40 years, (2) normal renal function at the time of death, and (3) Mayo imaging class 1A or 1B.

ADPKD, autosomal dominant polycystic kidney disease.

## Disclosure

All the authors declared no competing interests.

## Patient Consent

The authors declare consent was obtained from the patient discussed in the report.
